# Genetic characterization and zoonotic potential of *Blastocystis* from wild animals in Sichuan Wolong National Natural Reserve, Southwest China

**DOI:** 10.1051/parasite/2021071

**Published:** 2021-10-27

**Authors:** Shanyu Chen, Wanyu Meng, Ziyao Zhou, Lei Deng, Xiaogang Shi, Yijun Chai, Haifeng Liu, Yuehong Cheng, Zhijun Zhong, Hualin Fu, Liuhong Shen, Kun Zhang, Tingmei He, Guangneng Peng

**Affiliations:** 1 Key Laboratory of Animal Disease and Human Health of Sichuan Province, College of Veterinary Medicine, Sichuan Agricultural University Chengdu 611130 Sichuan PR China; 2 Sichuan Wolong National Natural Reserve Administration Aba 623006 Sichuan PR China

**Keywords:** *Blastocystis*, Wild animals, Prevalence, Subtypes, China

## Abstract

*Blastocystis* is a prevalent eukaryotic parasite that has been identified in a wide range of hosts. Several species are considered potential sources of *Blastocystis* infection in humans, but little is known about the prevalence of *Blastocystis* in wild animals. In this study, the prevalence and subtypes of *Blastocystis* were investigated to assess the zoonotic potential of wild animals in Sichuan Wolong National Natural Reserve. A total of 300 fecal samples were collected from 27 wildlife species in three areas of the Reserve. The subtype (ST), genetic characteristics, and prevalence of *Blastocystis* were determined by PCR amplification of part (~600 bp) of the SSU rRNA gene. Thirty fecal samples (10.0%) were *Blastocystis*-positive. The highest prevalence of *Blastocystis* was found in Yinchanggou (18.3%), with significantly less found in Niutoushan (7.5%) and Genda (5.5%) (*p* < 0.05). No significant differences were associated with different orders of animals in prevalence, which may be because of the small number of positive samples obtained. Sequence analysis showed five subtypes (ST1, ST3, ST5, ST13, and ST14), with ST13 and ST14 being predominant (33% each), followed by ST1 (20%). This is the first molecular investigation of *Blastocystis* infection in the wild animals of southwestern China. Subtypes ST1, ST3, ST5, and ST14 have previously been identified in humans, suggesting that wild animals may be potential reservoirs of *Blastocystis* for humans.

## Abstract (fr)

*Blastocystis* est un parasite eucaryote répandu qui a été identifié dans un large éventail d’hôtes. Plusieurs espèces sont considérées comme des sources potentielles d’infection par *Blastocystis* chez l’homme, mais on sait peu de choses sur la prévalence de *Blastocystis* chez les animaux sauvages. Dans cette étude, la prévalence et les sous-types de *Blastocystis* ont été étudiés pour évaluer le potentiel zoonotique des animaux sauvages dans la réserve naturelle nationale du Sichuan Wolong. Au total, 300 échantillons de matières fécales ont été prélevés de 27 espèces de la faune dans trois zones de la réserve. Le sous-type (ST), les caractéristiques génétiques et la prévalence de *Blastocystis* ont été déterminés par amplification PCR d’une partie (~ 600 pb) du gène 18S de l’ARNr. Trente échantillons fécaux (10,0 %) étaient positifs pour *Blastocystis*. La prévalence la plus élevée de *Blastocystis* a été trouvée à Yinchanggou (18,3 %), avec une prévalence significativement moindre à Niutoushan (7,5 %) et à Genda (5,5 %) (*p* < 0,05). Aucune différence significative de prévalence n’était associée à différents ordres d’animaux, ce qui peut être dû au petit nombre d’échantillons positifs obtenus. L’analyse des séquences a montré cinq sous-types (ST1, ST3, ST5, ST13 et ST14), et ST13 et ST14 étant prédominants (33 % chacun), suivis de ST1 (20 %). Il s’agit de la première étude moléculaire de l’infection à *Blastocystis* chez les animaux sauvages du sud-ouest de la Chine. Les sous-types ST1, ST3, ST5 et ST14 ont déjà été identifiés chez l’homme, suggérant que les animaux sauvages pourraient être des réservoirs potentiels de *Blastocystis* pour l’homme.

## Introduction

The enteric parasite *Blastocystis* (classified as a Stramenopile) is the most common protist found in humans [34]. The primary mode of transmission is through *Blastocystis*-contaminated water and food via the fecal-oral route [13]. There is strong evidence to suggest that some human infections may be caused by the zoonotic transmission of *Blastocystis* [25, 36]. The pathogenicity of *Blastocystis* remains controversial, with studies associating it with various gastrointestinal disorders such as inflammatory bowel disease (IBD) and irritable bowel syndrome (IBS) [9, 16, 18]. However, the few microbiome studies on this organism have indicated that *Blastocystis* is a common commensal in the human gut and is associated with increased bacterial diversity [6].

Based on sequence analysis of the small subunit ribosomal (SSU) RNA gene, at least 22 subtypes of *Blastocystis* have been identified in animals and humans worldwide [31]. Subtypes ST1–10, ST12, and ST14 have been found in humans with varying prevalence, but ST1–4 are the most common, accounting for more than 90% of human *Blastocystis* infections [20, 26, 33]. Accumulating evidence has shown that the same subtype of *Blastocystis* can colonize a wide range of hosts, implying that these subtypes lack host-specific features.

In China, *Blastocystis* has been found in humans and both domestic and captive wildlife animals belonging to the orders Carnivora, Artiodactyla, Perissodactyla, Rodentia, and primates [10, 13–15, 37, 43], highlighting these animals as potential hosts for human infection with *Blastocystis*. However, limited studies have been conducted in which *Blastocystis* has been isolated from wild animals in China, and their role as a reservoir of infection for humans and other animals remains unknown.

Sichuan Wolong National Natural Reserve is the third largest nature reserve in China, covering an area of 200,000 ha. The area is known for its complex natural conditions and is inhabited by the largest number of rare animals in Sichuan Province (https://baike.so.com/doc/5376249-5612365.html). The connection with the outside world via the development of tourism means that the chances of contact between animals and humans in the reserve have greatly increased, heightening the risk of zoonotic transmission. Therefore, the purpose of this study was to investigate the prevalence and subtypes of *Blastocystis* in the wild animals of this reserve and to assess the zoonotic potential of the *Blastocystis* colonizing these animals.

## Materials and methods

### Ethical statement

This study was performed in accordance with the recommendations of the Guide for the Care and Use of Laboratory Animals of the Ministry of Health, China. Only fecal samples collected after spontaneous defecation of the wild animals were analyzed. Consequently, this study did not require full Animal Ethics Committee approval, in accordance with Chinese law. No animals were harmed during the sampling process. Permission was obtained from reserve managers prior to collection of fecal specimens.

### Sample collection

**Figure 1:**
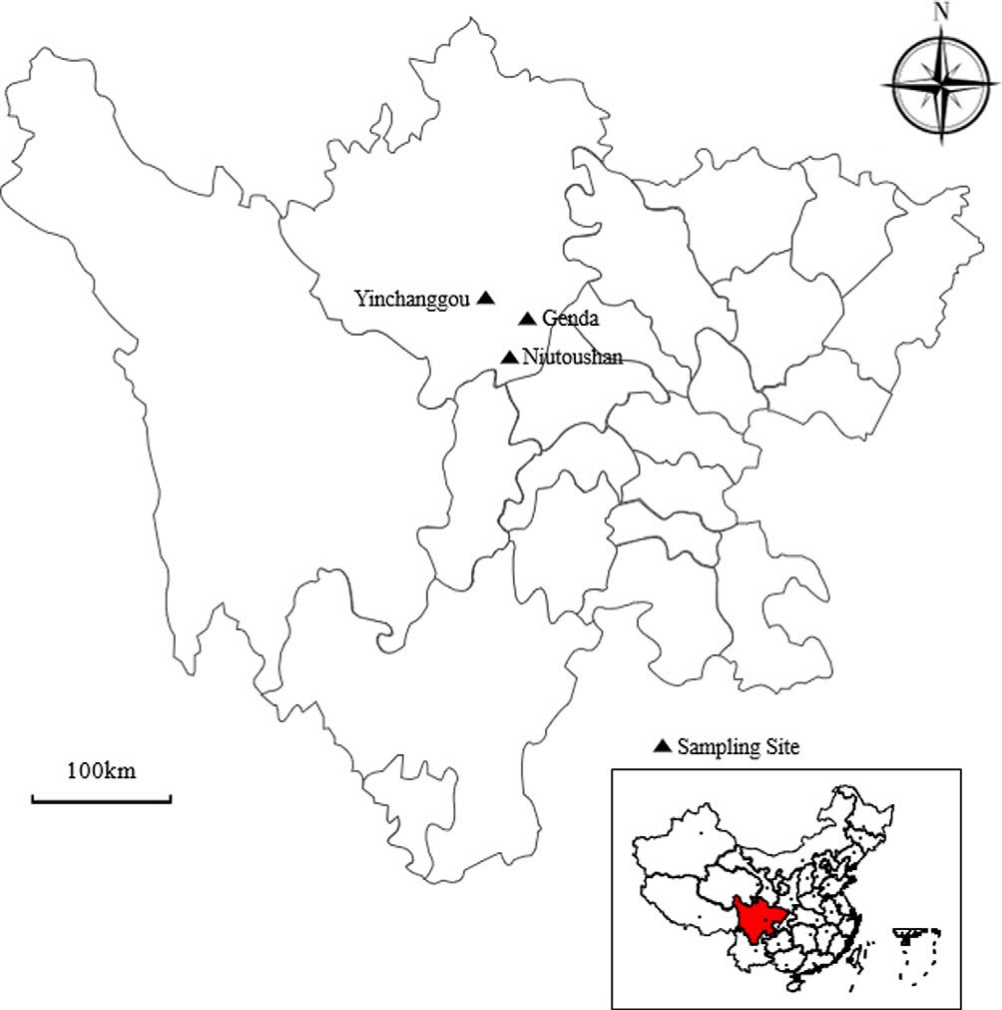
Geographical distribution of the sampled sites (filled triangle) in Sichuan Province, Southwestern China.

A total of 300 fecal samples was collected from three areas within the Sichuan Wolong National Natural Reserve (Fig. 1) between March 2020 and December 2020, with 127 samples collected in Genda, 93 in Yinchanggou, and 80 in Niutoushan (Tables 1 and 2). All fecal samples were collected by experienced mountain patrol staff of the Sichuan Wolong National Natural Reserve during patrols and strict controls were implemented to minimize potential contamination between samples from different animal species. Camouflaged video equipment was placed to identify the species of animal prior to sampling, based on its range or nesting location. Briefly, stool samples were collected from tracks or in the vicinity of nests. The species from which a sample originated was inferred in the field according to the shape, size, and texture of the fecal sample, footprints, and the presence of nearby nests, and confirmed with video information. Some animal feces were collected immediately after the animal was observed to defecate, including Galliformes, primates, and some Artiodactyla. Fecal samples for which the species could not be identified or samples that were older than two days were not included. All fecal samples were collected in sterilized plastic containers using disposable sterile gloves and preserved at 4 °C until DNA extraction.

**Table 1:** Factors associated with the prevalence of *Blastocystis* in wild animals in China.

Factor	No. positive/overall	Prevalence (95% CI)	OR (95% CI)	*p* value
Locations				
Genda	7/127	5.5 (1.5–9.5)	Reference	Reference
Yinchanggou	17/93	18.3 (10.4–26.1)	3.8 (1.5–9.7)	0.004
Niutoushan	6/80	7.5 (1.7–13.3)	1.4 (0.5–4.3)	0.6
Host				
Rodentia	1/7	14.3 (11.6–40.2)	Reference	Reference
Primates	1/5	20.0 (15.1–55.1)	1.5 (0.1–31.6)	0.8
Artiodactyla	26/198	13.1 (8.4–17.8)	0.9 (0.1–7.8)	0.9
Carnivora	2/87	2.3 (0.9–5.4)	0.1 (0.01–1.8)	0.1
Galliformes	0/3	0.0	0 (0)	1.0
Total	30/300	10.0 (6.6–13.4)		

**Table 2:** Animal samples collected from various hosts from three different areas in Sichuan Wolong National Nature reserve in Sichuan Province, southwestern China.

Host	Scientific name	GD	YC	NT	No. of *Blastocystis*-positive/overall
Primates					
Tibetan macaque	*Macaca thibetana*		3		0/3
Snub-nosed monkey	*Rhinopithecus roxellanae*	2			1/2
Artiodactyla					
Sambar	*Rusa unicolor*	18	13	8	8/39
Sika deer	*Cervus nippon*		14		5/14
Long-tailed goral	*Naemorhedus griseus*		15	2	5/17
Crested deer	*Elaphodus cephalophus*	2	4	2	4/8
Chinese antelope	*Capricornis milneedwardsii*	1	1	1	1/3
Dwarf musk deer	*Moschus berezovskii*	12	1	6	0/19
Takin	*Budorcas taxicolor*	15	9	1	0/25
Blue sheep	*Pseudois nayaur*	34		8	3/42
Tibetan antelope	*Pantholops hodgsonii*			1	0/1
Yak	*Bos mutus*			15	0/15
Gaur	*Bos gaurus*	1			0/1
Goral	*Naemorhedus goral*	10			0/10
Wild pig	*Sus scrofa*	4			0/4
Rodentia					
Porcupine	*Hystrix hodgsoni*	5	1	1	1/7
Carnivora					
Sand badger	*Arctonyx collaris*		2	1	1/3
Lesser panda	*Ailurus fulgens*	2	8		0/10
Leopard cat	*Prionailurus bengalensis*	3	3	4	1/10
Giant panda	*Ailuropoda melanoleuca*	11	19	10	0/40
Stone Marten	*Martes foina*			2	0/2
Swinhoe	*Paguma larvata taivana*			1	0/1
Asiatic black bear	*Ursus thibetanus*			1	0/1
Jackal	*Cuon alpinus*	1			0/1
Snow leopard	*Panthera uncia*	6		13	0/19
Galliformes					
Blood Pheasant	*Ithaginis cruentus*			1	0/1
Phasianus versicolor	*Lophophorus lhuysii*			2	0/2
Total		127	93	80	30/300

GD = Genda; YC = Yinchanggou; NT Niutoushan.

### DNA extraction

All fecal specimens were sieved and washed three times with distilled water by centrifugation at 3000 ×*g* for 10 min. Genomic DNA was extracted using a QIAamp DNA Stool Mini Kit (Qiagen, Hilden, Germany) from approximately 250 mg of sample, according to the manufacturer’s instructions. Both *Blastocystis-*positive and negative fecal sample controls were included. The quality of the DNA was verified using NanoDrop (Thermo Fisher Scientific, Carlsbad, CA, USA) and the DNA was eluted in 50 μL of nuclease-free water and stored at −20 °C until PCR analysis.

### Polymerase chain reaction (PCR) amplification

PCR amplification of the barcode region (∼600 bp) of the SSU rRNA gene was used to screen all DNA preparations for the presence of *Blastocystis* with the primers and cycling parameters previously described by Scicluna et al. [27]. The Taq PCR Master Mix (Sangon Biotech Co., Ltd., Shanghai, China) was used for all PCRs. Reagents used per 25 μL reaction were as follows: 12.5 μL Taq PCR Master Mix (Sangon Biotech Co., Ltd., Shanghai, China), 1 μL of each primer (0.4 μM), 2 μL of genomic DNA sample, 1.5 mM MgCl_2_, and nuclease-free water to the desired volume. All PCR tests were performed in triplicate, and both positive and negative controls were included. PCR products were subjected to 1.5% agarose gel electrophoresis (AddGene, Watertown, MA, USA) and visualized by staining with SYBR Safe DNA Gel Stain (Thermo Fisher Scientific).

### Nucleotide sequencing and analysis

PCR products with the predicted size (approximately 600 bp) were excised from the agarose gel and purified using a QIAquick Gel Extraction Kit (Qiagen), according to the manufacturer’s instructions. All PCR-positive products were bidirectionally sequenced on an ABI PRISMTM 3730 DNA Analyzer (Applied Biosystems, Foster City, CA, USA), using a BigDye Terminator v3.1 Cycle Sequencing kit (Applied Biosystems). The nucleotide sequences obtained were subjected to BLAST searches (http://www.ncbi.nlm.nih.gov/blast/) and then aligned and analyzed. Reference sequences were downloaded from the GenBank database using the program Clustal X 2.0 (http://www.clustal.org/) to determine the subtypes of the *Blastocystis* isolates. The representative nucleotide sequences generated were deposited in GenBank under the accession numbers MW404496, MW404497, MW404561, MW404583, MW404585, MW404588, and MW404590.

### Phylogenetic analysis

To assess the genetic relationships of the *Blastocystis* genotypes in this study with sequences from GenBank that were identified in previous studies, phylogenetic analysis was performed by constructing a neighbor-joining tree using MEGA 6 software (http://www.megasoftware.net/). The evolutionary distances were calculated using the Kimura 2-parameter model. Undefined positions were removed from the alignment prior to phylogenetic analysis, and the alignment was trimmed using MEGA 6 (http://www.megasoftware.net/). The reliability of the trees was assessed by bootstrap analysis with 1000 replicates.

### Statistical analysis

The prevalence of *Blastocystis* in the different study areas and the orders of animals infected were analyzed with the binary logit model using SPSS 22 (https://www.ibm.com/analytics/spss-statistics-software). Each of these variables was included in the binary logit model as an independent variable via multivariable regression analysis. The results were considered statistically significant when the *p*-value was < 0.05. The adjusted odds ratio (OR) and 95% confidence interval (CI) for each variable were calculated using binary logistic regression, and all risk factors were entered simultaneously.

## Results

### Prevalence of *Blastocystis* in wild animals

In the present study, 30 of the 300 fecal samples (10.0%) collected from three areas of Sichuan Wolong National Natural Reserve, southwestern China, were determined to be *Blastocystis*-positive by PCR amplification of the barcode region of the SSU rRNA gene. The highest prevalence of *Blastocystis* was observed in Yinchanggou, followed by Niutoushan and Genda (Table 1). The difference in *Blastocystis* prevalence was significant in the three areas (*p* < 0.05). However, the difference in *Blastocystis* prevalence among animals of different orders was not significant (*p* > 0.05).

Overall, of the 27 species tested in this study, 10 (37.0%) were positive for *Blastocystis* (Table 2). Of the 16 species tested in Genda, 4 (25.0%) were positive for *Blastocystis*. The prevalence of the parasite was 53.8% (7/13) in Yinchanggou and 21.1% (4/19) in Niutoushan.

### Subtype distributions of *Blastocystis* in wild animals

Five subtypes of *Blastocystis* were identified among the 30 positive samples, including three potentially zoonotic STs (ST1, ST3, and ST5) and two animal-specific STs (ST13 and ST14). ST13 (10/30) and ST14 (10/30) were the dominant subtypes found in the wild animals examined (Table 3), followed by ST1 (6/30), ST5 (3/30), and ST3 (1/30).

**Table 3:** Prevalence of *Blastocystis* among different species.

Species	Prevalence (No. of positive/overall)	GD	YC	NT
Snub-nosed monkey	50.0 (1/2)	ST5 (1)		
Sambar	20.5 (8/39)	ST13 (3)	ST14 (3)	ST14 (2)
Sika deer	35.7 (5/14)		ST1 (4); ST3 (1)	
Long-tailed goral	29.4 (5/17)		ST1 (1); ST5 (2); ST13 (2)	
Crested deer	50.0 (4/8)	ST13 (1)	ST13 (1)	ST13 (1); ST14 (1)
Chinese antelope	33.3 (1/3)		ST14 (1)	
Blue sheep	7.1 (3/42)	ST13 (2)		ST14 (1)
Porcupine	14.3 (1/7)		ST1 (1)	
Sand badger	33.3 (1/3)			ST14 (1)
Leopard cat	10.0 (1/10)		ST14 (1)	
Total	20.7 (30/145)	ST5 (1); ST13 (6)	ST1 (6); ST3 (1); ST5 (2); ST13 (3); ST14 (5)	ST13 (1); ST14 (5)

GD = Genda; YC = Yinchanggou; NT = Niutoushan.

### Genetic characteristics of *Blastocystis* subtypes

Identity analysis of the SSU rRNA gene revealed six sequences of ST1 isolate in sika deer, long-tailed goral, and porcupine that were identical to those found in chimpanzees from Tanzania: Rubondo Island (HQ286905). Similarly, one ST3 sequence found in sika deer showed 100% identity with the GenBank sequence MW242639 (from the red-bellied tree squirrel in China), and three ST5 sequences had 100% similarity with that of sheep in China: Heilongjiang (MF974615).

Ten ST13 isolates contained two representative sequences. The sequence MW404585, which was found in ST13 isolates obtained from long-tailed goral, crested deer, and blue sheep, showed 99.43% similarity with a ST13 sequence that was isolated from a reindeer in China (MH325366), with only three nucleotide substitutions. The remaining sequence, MW404588, which was obtained from sambar and long-tailed goral, showed 100% similarity with an ST13 sequence MF186700 from a muntjac. Similarly, two representative sequences were also obtained in 10 of the ST14 isolates. The sequences MW404561 (obtained from sambar, Chinese antelope, and sand badger) and MW404583 (obtained from sambar, crested deer, blue sheep, and leopard cat) showed 99.42% identity with an ST14 sequence that was previously isolated from a sheep in the Czech Republic (MT039559) and 99.81% similarity with that of an isolate from sika deer in China (MK357783), with three and one nucleotide substitutions, respectively.

### Phylogenetic analysis of *Blastocystis*

**Figure 2:**
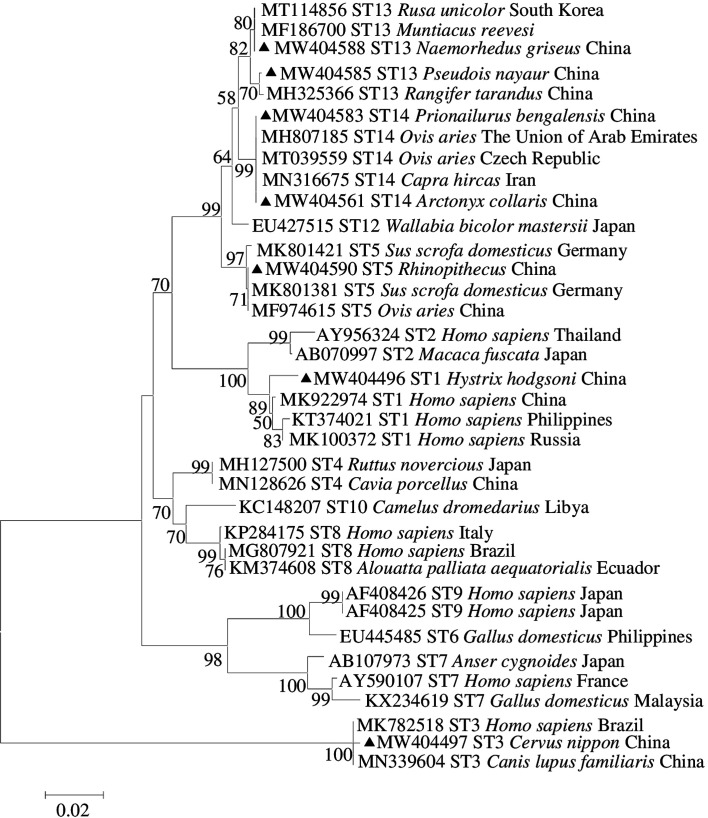
Phylogenetic relationships among nucleotide sequences of *Blastocystis* partial small subunit ribosomal RNA (SSU rRNA) genes. The neighbor-joining method was used to construct the trees by the Kimura-2-parameter model. The numbers on the branches are percent bootstrapping values from 1000 replicates, with values of more than 50% shown in the tree. Each sequence is identified by its accession number, subtypes, host origin, and country. Genotypes marked with black triangles are known genotypes identified in this study, respectively.

Seven representative sequences were obtained from the 30 *Blastocystis* isolates in this study. These sequences showed high identity with reference sequences for *Blastocystis* in GenBank. Newly acquired sequences belonged to ST1, ST3, ST5, ST13, and ST14. ST1 clustered with sequences from humans from the Philippines, Russia, and China. ST3 can be grouped with sequences that are mainly from humans in Brazil and dogs in China. ST5 clustered with sequences from sheep in China and pigs in Germany, while ST13 can be grouped with sequences isolated from reindeer muntjac and sambar. ST14 forms a clade with sequences from cattle, sheep, and goats (Fig. 2).

## Discussion

*Blastocystis* is the most frequent parasite reported in humans and several other animals with controversial pathogenicity [24, 41]. Previous studies have linked infection with *Blastocystis* to nutritional and gastrointestinal disorders in both developing and developed countries [28]. However, recent microbiome studies have reported that *Blastocystis* may be an indicator of good intestinal health [5]. Zoonotic STs are occasionally transmitted between animals and humans, and some animals may be significant potential reservoirs for human infection [22, 29, 44].

Epidemiological studies have been conducted in domestic animals, including pigs, cattle, sheep, and goats, but only a few reports on captive wildlife have been documented in China [13]. The prevalence of *Blastocystis* in the wild animals examined in this study was 10.0% (30/300); this is lower than that previously found in zoo animals in Western Australia (42%, 32/76) [25], wild animals on Qinling Mountains, China (40.2%, 200/497) [42], zoo animals in Japan (39.0%, 46/118) [2], wild animals in Brazil (34.4%, 115/334) [35], zoo animals in the United Kingdom (34.2%, 79/231), various captive animals in France (32.2%, 99/307) [12], captive wildlife in four zoos of southwestern China (15.7%, 66/420) [15], and captive mammalian wildlife in Bangladesh National Zoo (15.5%, 31/200) [29]. However, the prevalence is higher than that observed in animals from three city zoos in China (6.0%, 27/450) [23]. At this time, it is unclear how factors such as the conditions in which animals are housed, the animal species sampled, the number of samples examined, and management methods affect the prevalence, both in different countries and in the same country.

The *Blastocystis* prevalence of 20.0% found in non-human primates (NHPs) was higher than that in rodents (14.3%), Artiodactyla (13.1%), Carnivora (2.3%), and Galliformes (0.0%). However, the small number of positive samples meant that the differences observed between these groups were not significant. Five *Blastocystis* STs, including ST1, ST3, ST5, ST13, and ST14, were identified in 30 *Blastocystis*-positive samples from four orders of wildlife (Tables 2 and 3). These STs have also been documented in European [8, 12, 27, 32] and Brazilian primates [35], while ST4, ST5, and ST8 have previously been found in monkeys [27, 32, 35, 40]. The sequences obtained from primates in this study belonged to ST5, infecting the snub-nosed monkey. ST5 has also been found sporadically in humans that are in close contact with animals, suggesting zoonotic transmission [30, 39].

Previous studies have confirmed the presence of other STs such as ST1-3, ST5, ST7, ST8, ST10, and ST17 in rodents [4, 7]. In this study, ST1 was identified in rodents (porcupine), corroborating the previous data. The infection of Wistar rats with ST1 has been reported to lead to moderate and severe pathological changes, indicating the potential pathogenicity of this subtype [17].

Five subtypes (ST1, ST3, ST5, ST13, and ST14) were identified in Artiodactyla (long-tailed goral, sika deer, sambar, crested deer, Chinese antelope, and blue sheep). Previous studies have reported that many animals in the order Artiodactyla harbor *Blastocystis*, including cattle, pigs, sheep, deer, and goats [13, 19]. To date, most STs, including ST1, ST3, ST5, ST13, and ST14, have been identified in Artiodactyla [4, 38]. In this study, ST1 was found in sika deer and long-tailed goral, ST3 was identified in sika deer, ST5 was found in long-tailed goral, ST13 was found in sambar, long-tailed goral, crested deer, and blue sheep, and ST14 was identified in sambar, crested deer, Chinese antelope, and blue sheep. Many previous surveys have indicated that ST1 and ST3 are the two most common subtypes infecting humans in several countries [1, 3]. This study has identified the potential for transmission of *Blastocystis* infection between humans and Artiodactyla. Therefore, the role of Artiodactyla in transmitting these subtypes should be further evaluated in future studies. ST5 has been identified in various animals, such as cattle, sheep, NHPs, and birds [19]. Surprisingly, ST13 was also determined in Java mouse-deer in France [12] and in a mouse deer in the United Kingdom [4]. Meanwhile, sheep in China [21] and muntjac deer in the United Kingdom have also been found to be infected with ST14 [7].

The sequences obtained from Carnivora in this study belonged to ST14 with the isolates infecting sand badgers and leopard cats. ST14 is often reported in artiodactyls but is almost absent in Carnivora [7].

## Conclusions

To the best of our knowledge, this is the first molecular investigation in which *Blastocystis* infection has been observed in sambar, long-tailed goral, crested deer, Chinese antelope, blue sheep, sand badger, and leopard cat in China, further broadening the host range of *Blastocystis* [11]. The prevalence of *Blastocystis* was 10.0% (30/300) in the wild animals of southwestern China, with five *Blastocystis* subtypes (ST1, ST3, ST5, ST13, and ST14) identified in this study. ST1, ST3, and ST5 are considered zoonotic subtypes, suggesting that these wild animals may serve as natural reservoirs for human *Blastocystis* infection. The findings of the present study provide preliminary data for monitoring and investigating the transmission routes of *Blastocystis*.

## Statement

The study has been published as a preprint.

## Abbreviations

STs: Subtypes
PCR: Polymerase chain reaction
SSU rRNA: Small subunit ribosomal RNA
IBD: Inflammatory bowel disease
IBS: Irritable bowel syndrome
ORs: Odds ratios
NHPs: Nonhuman primates

## Authors’ contributions

This study was conceived and designed by GP. Experiments were performed by SC, WM, TH, ZZ (Ziyao Zhou), and LD. Fecal samples were collected by XS, YC, HL, YC, ZZ (Zhijun Zhong), and HF. Data were analyzed by LS and KZ. All authors have read and approved the submitted version of this manuscript.

## Funding

This work was funded by the Special Fund for Forestry Reform and Development of Wolong Special Administrative Region of Sichuan Province (510000-02-064387).

## Availability of data and materials

The nucleotide sequences generated in the present study have been deposited in GenBank (https://www.ncbi.nlm.nih.gov/) under accession numbers MW404496, MW404497, MW404561, MW404583, MW404585, MW404588, and MW404590. The datasets used and/or analyzed during the current study are available from the corresponding author on reasonable request.

## Competing interests

The authors declare that they have no competing interests
